# Differences between murine arylamine *N*-acetyltransferase type 1 and human arylamine *N*-acetyltransferase type 2 defined by substrate specificity and inhibitor binding

**DOI:** 10.1186/2050-6511-15-68

**Published:** 2014-11-29

**Authors:** Nicola Laurieri, Akane Kawamura, Isaac M Westwood, Amy Varney, Elizabeth Morris, Angela J Russell, Lesley A Stanley, Edith Sim

**Affiliations:** Department of Pharmacology, University of Oxford, Oxford, UK; Department of Chemistry, Chemistry Research Laboratory, University of Oxford, Oxford, UK; Divisions of Structural Biology and Cancer Therapeutics, The Institute of Cancer Research, Chester Beatty Laboratories, London, UK; Consultant in Investigative Toxicology, Linlithgow, West Lothian, Scotland; Faculty of Science, Engineering and Computing, Kingston University, Kingston on Thames, UK

**Keywords:** Arylamine *N*-acetyltransferase, (MOUSE)NAT1, (MOUSE)NAT2, Substrate specificity, Inhibitor selectivity, Structural docking

## Abstract

**Background:**

The mouse has three arylamine *N*-acetyltransferase genes, *(MOUSE)Nat1*, *(MOUSE)Nat2* and *(MOUSE)Nat3*. These are believed to correspond to *(HUMAN)NAT1*, *(HUMAN)NAT2* and *NATP* in humans. *(MOUSE)Nat3* encodes an enzyme with poor activity and human *NATP* is a pseudogene. *(MOUSE)Nat2* is orthologous to *(HUMAN)NAT1* and their corresponding proteins are functionally similar, but the relationship between *(MOUSE)Nat1* and *(HUMAN)NAT2* is less clear-cut.

**Methods:**

To determine whether the (MOUSE)NAT1 and (HUMAN)NAT2 enzymes are functionally equivalent, we expressed and purified (MOUSE)NAT1*1 and analysed its substrate specificity using a panel of arylamines and hydrazines. To understand how specific residues contribute to substrate selectivity, three site-directed mutants of (MOUSE)NAT2*1 were prepared: these were (MOUSE)NAT2_F125S, (MOUSE)NAT2_R127G and (MOUSE)NAT2_R127L. All three exhibited diminished activity towards “(MOUSE)NAT2-specific” arylamines but were more active against hydrazines than (MOUSE)NAT1*1. The inhibitory and colorimetric properties of a selective naphthoquinone inhibitor of (HUMAN)NAT1 and (MOUSE)NAT2 were investigated.

**Results:**

Comparing (MOUSE)NAT1*1 with other mammalian NAT enzymes demonstrated that the substrate profiles of (MOUSE)NAT1 and (HUMAN)NAT2 are less similar than previously believed. Three key residues (F125, R127 and Y129) in (HUMAN)NAT1*4 and (MOUSE)NAT2*1 were required for enzyme inhibition and the associated colour change on naphthoquinone binding. *In silico* modelling of selective ligands into the appropriate NAT active sites further implicated these residues in substrate and inhibitor specificity in mouse and human NAT isoenzymes.

**Conclusions:**

Three non-catalytic residues within (HUMAN)NAT1*4 (F125, R127 and Y129) contribute both to substrate recognition and inhibitor binding by participating in distinctive intermolecular interactions and maintaining the steric conformation of the catalytic pocket. These active site residues contribute to the definition of substrate and inhibitor selectivity, an understanding of which is essential for facilitating the design of second generation (HUMAN)NAT1-selective inhibitors for diagnostic, prognostic and therapeutic purposes. In particular, since the expression of (HUMAN)NAT1 is related to the development and progression of oestrogen-receptor-positive breast cancer, these structure-based tools will facilitate the ongoing design of candidate compounds for use in (HUMAN)NAT1-positive breast tumours**.**

**Electronic supplementary material:**

The online version of this article (doi:10.1186/2050-6511-15-68) contains supplementary material, which is available to authorized users.

## Background

Arylamine *N-*acetyltransferases (NATs, EC. 2.3.1.5) [1] are drug metabolising enzymes which catalyse the conjugation of an acetyl group from acetyl Coenzyme A (AcCoA) to arylamines, hydrazines and *N-*hydroxyarylamines. They participate in the detoxification and metabolic activation of xenobiotics and are found in a wide variety of prokaryotic and eukaryotic species [2].

The human genome contains two polymorphic *NAT* genes, *(HUMAN)NAT1* and *(HUMAN)NAT2* [3, 4], which play important pharmacogenetic roles in cancer susceptibility and have the potential to contribute to personalised medicine [5, 6]. The corresponding enzymes possess distinct substrate profiles: HUMAN(NAT1) preferentially *N-*acetylates the arylamines 4-aminobenzoic acid (4ABA), 4-aminobenzoyl glutamate (4ABglu) and 4-aminosalicylate (4AS), whereas (HUMAN)NAT2 has higher activity towards the hydrazines isoniazid (INH) and hydralazine (HDZ) and the arylamine sulfamethazine (SMZ) [7, 8]. The crystal structures of human NATs [9] resemble the three-domain conformation observed in prokaryotic NATs [10]. Like their prokaryotic counterparts, each has a catalytic triad comprising cysteine (C), histidine (H) and aspartic acid (D) at the active site; they also contain an additional loop of 17 residues which many prokaryotic enzymes lack. These observations helped to lay the foundations for studies addressing the structural determinants of their catalytic selectivity [11].

Human NATs are characterised by differing tissue distributions and patterns of gene expression during development [12–14]. In particular, while (HUMAN)NAT2 appears to be a well-established drug metabolising enzyme and is mainly expressed in the liver [15], the selective *N*-acetylation of the folate catabolites 4ABglu and 4ABA by (HUMAN)NAT1 suggests that this isoform may have an endogenous role in folate homeostasis [16, 17]. Furthermore, recent studies indicate that (HUMAN)NAT1 has a novel catalytic function as a folate-dependent AcCoA hydrolase [18].

Rodents also have multiple NAT isoforms which have been investigated as animal models for the corresponding human enzymes. Mice, for example, have three *NAT* genes, *(MOUSE)Nat1*, *(MOUSE)Nat2* and *(MOUSE)Nat3*. All of these are polymorphic; the *(MOUSE)Nat3* gene exhibits the greatest extent of polymorphism and may effectively be considered a pseudogene like *(HUMAN)NAT3* [19], although it does have enzymatic activity [20, 21].

Historically, (MOUSE)NAT2 has been considered to correspond to (HUMAN)NAT1 because their primary sequences are 82% homologous and they exhibit similarities in terms of expression profile, tissue distribution and substrate preference [8, 22–24]. Furthermore, inhibition studies have identified a (HUMAN)NAT1/(MOUSE)NAT2 selective inhibitor, naphthoquinone **1** (*N*-(3-(3,5-dimethylphenylamino)-1,4-dioxo-1,4-dihydronaphthalen-2-yl)benzenesulphonamide; Additional file 1: Figure S1), which undergoes a distinctive colour change from red to blue upon binding to either of these two enzymes but not to other mammalian NAT isoenzymes [24, 25]. Virtual modelling studies [25, 26] indicate that the interaction between naphthoquinone **1** and (HUMAN)NAT1 or (MOUSE)NAT2 depends on selective ionic interactions between the conjugate base of compound **1** and the guanidinium moiety of an active site residue, arginine 127 (R127). This is part of a group of active site residues which had previously been identified as being involved, together with phenylalanine 125 (F125) and tyrosine 129 (Y129), in substrate specificity [27, 28]. These residues differ between (HUMAN)NAT1*4 and (HUMAN)NAT2*4 and between (MOUSE)NAT1*1 and (MOUSE)NAT2*1, while the catalytic triad of C, H and D is identical.

The (MOUSE)NAT1 enzyme is commonly assumed to be functionally equivalent to (HUMAN)NAT2 because both can metabolise isoniazid (INH) and sulfamethazine (SMZ) [29–31]. In order to determine whether these enzymes are true functional equivalents, we used a previously reported (MOUSE)Nat1*1 clone [32] to express and purify the (MOUSE)NAT1*1 enzyme and compared its substrate profile with those of other rodent and human NAT enzymes using a broad panel of aromatic amines and hydrazines. In addition, we used three site-directed mutants ((MOUSE)NAT2_F125S, (MOUSE)NAT2_R127G and (MOUSE)NAT2_R127L) to investigate the effects of key active site residues on the substrate specificity of (MOUSE)NAT2. In the present study we focused on residues 125 and 127; the role of the Y129 residue found in (HUMAN)NAT1 and (MOUSE)NAT2, at least with respect to inhibitor binding, has previously been investigated using (MESAU)NAT2, which has identical active site residues except for a leucine (L) at location 129 [33]. The results of this earlier study [26] suggest that Y129 is functionally important, at least in inhibitor recognition, and illustrate the value of (MESAU)NAT2 as a protein model for comparative studies with (HUMAN)NAT1 and (MOUSE)NAT2.

Finally, we modelled the binding of representative arylamine substrates within the active sites of reference and mutant mammalian NATs in order to elucidate key interactions within the NAT/substrate complex. The identification of (HUMAN)NAT1 as a potential therapeutic target in cancer means that understanding the molecular details of this series of enzymes in humans and potential animal models is important for their potential exploitation in both diagnostics [24] and therapy [34].

## Methods

### Chemicals and reagents

All chemicals were purchased from Sigma-Aldrich unless otherwise stated. Molecular biology reagents were obtained from Promega.

### Expression of pure recombinant NATs

#### Expression and purification of (MOUSE)NAT1*1

The open reading frame of (MOUSE)Nat1*1 [32] was subcloned into the *Nde*I and *Eco*RI sites of pET28b(+) (Novagen) and transformed into *Escherichia coli* BL21(DE3)CodonPlus-RIL (Stratagene). BL21 cells carrying the expression plasmid were grown in auto-induction medium at 27°C in the presence of kanamycin (30 μg.mL^−1^) and chloramphenicol (34 μg.mL^−1^) and harvested after 24 hrs by centrifugation (5,000 g, 4°C, 20 min). The cell pellet was resuspended in lysis buffer (300 mM NaCl, 20 mM Tris–HCl (pH 8.0), 1 × EDTA-free Complete Protease Inhibitor (Roche)) and stored at −80°C. Cells were thawed, lysed by sonication and the soluble protein fraction was separated from cell debris by centrifugation (12,000 *g*, 4°C, 20 min). The soluble fraction was incubated with pre-equilibrated Ni-NTA resin (Qiagen) for 5 min, loaded on a chromatography column and washed with buffer solutions containing increasing imidazole concentrations at 4°C (two washes each of 0 mM, 10 mM, 20 mM, 50 mM and 100 mM imidazole). Fractions containing (MOUSE)NAT1*1 were identified by sodium dodecyl sulphate-polyacrylamide gel electrophoresis and NAT activity assays using 5-aminosalicylate (5AS) [35]. The hexa-His tag was removed by thrombin cleavage (5 U thrombin/mg of protein, 16 h incubation, 4°C) and the cleaved protein was dialysed against 20 mM Tris–HCl (pH 8.0), 1 mM DTT, 1 mM EDTA buffer. Glycerol was added to 5%. Samples were concentrated by centrifugal ultrafiltration (Amicon), snap frozen in liquid nitrogen and stored at −80°C (1 mg.mL^−1^ in 20 mM Tris–HCl (pH 7.0), 5 mM DTT, 5% glycerol).

#### Site-directed mutagenesis, expression and purification of (MOUSE)NAT2_F125S

QuikChange II (Stratagene) was used to mutate codon 125 (TTT; F125) of (MOUSE)Nat2*1 [22] to TCT, which encodes serine (F125S) (Additional file 2: Figure S2). The resulting mutant was expressed in Rosetta(DE3)pLysS as described previously [22, 25]. (MOUSE)NAT2_F125S was purified by Ni-NTA affinity chromatography and thrombin cleavage, as described. Fractions containing mutant (MOUSE)NAT2_F125S protein were identified by sodium dodecyl sulphate-polyacrylamide gel electrophoresis and NAT activity assays using 4ABA [35].

#### Preparation of pure recombinant mammalian NATs

The preparation of pure recombinant (MOUSE)NAT2*1, (MOUSE)NAT2_R127G, (MOUSE)NAT2_R127L, (MESAU)NAT2*1, (HUMAN)NAT1*4 and (HUMAN)NAT2*4 was performed as described previously [8, 22, 25].

### Substrate and inhibitor selectivity and spectrometry of (MOUSE)NAT1*1 and three (MOUSE)NAT2 mutants

Substrate specific *N*-acetylation profiles were determined according to published methods with minor modifications [8, 36]. Recombinant proteins were used immediately after purification. The quantity of each protein used in assay mixes (100 μL) was: (MOUSE)NAT1*1, 0.633 μg; (MOUSE)NAT2*1, 1.4 μg; (MOUSE)NAT2_F125S, 1 μg; (MOUSE)NAT2_R127G, 0.8 μg; and (MOUSE)NAT2_R127L, 5.5 μg. The substrates used were 4ABglu, 4ABA, 4AS, 5AS, 4-chloroaniline (4CA), 4-bromoaniline (4BA), 4-iodoaniline (4IA), 4-methoxyaniline (ANS), 4-aminoveratrole (4AV), 4-hexyloxyaniline (HOA), 4-phenoxyaniline (POA), SMZ, INH and HDZ. In each case the final concentration in the reaction mix was 500 μM. All measurements were performed in triplicate and are expressed as mean ± standard deviation. For each enzyme tested, average measures of specific activity were calculated relative to the substrate exhibiting the highest specific activity (100%) for that enzyme.

In order to highlight differences in substrate preference among murine NAT proteins, the substrate profiles of (MOUSE)NAT2*1, (MOUSE)NAT1*1, (MOUSE)NAT2_F125S, (MOUSE)NAT2_R127G and (MOUSE)NAT2_R127L were compared by ANOVA (confidence limit 95%) using the Shapiro-Wilk test to verify normal distribution and Cochran’s test to check the equality of variances. Statistical significance was evaluated using Student’s t-test with a Bonferroni adjustment if required.

Inhibition of NAT activity was determined as the ratio of specific activity in the presence and absence of the inhibitor naphthoquinone **1** using the preferred substrate for the enzyme under investigation. The final concentration of dimethyl sulphoxide (DMSO) in reaction mixes was 5% (v/v). IC_50_ values were estimated using a dose–response-based regression model in Kyplot® software. Curves were fitted by least squares analysis with confidence limits of 95%. Visible spectra (λ = 800 to 326 nm) of naphthoquinone **1** in the presence of pure recombinant NATs were recorded with a U-2001 spectrophotometer (Hitachi) using 50 μL UVettes® (Eppendorf).

### Structural modelling and docking simulations

Structural models of wild type (MOUSE)NAT2*1, (MOUSE)NAT2 mutants and (MESAU)NAT2*1 were generated based on the structure of (HUMAN)NAT1*4 [PDB:2PQT] [9] using SwissModel software (http://swissmodel.expasy.org/) in automated mode [37–39]. Each substrate was drawn in 3D using ChemBio3D Ultra 12.0 and its ground state conformation predicted before it was docked into the catalytic pocket of the appropriate NAT structure ((HUMAN)NAT1*4: 2PQT, (HUMAN)NAT2*4: 2PFR [9]) or NAT model ((MOUSE)NAT2*1, (MOUSE)NAT2 mutants and (MESAU)NAT2*1). Protein and substrate structures were defined as pdbqt files and protein-structure interactions were analysed using Autodock Vina [40]. After adding polar hydrogen atoms to the NAT target and defining the rotatable bonds of the ligand, a docking site was defined within the active site pocket and possible solutions were ranked according to affinity energy (lowest to highest; kcal.mol^−1^). The docking results were visualised in 3D using PyMOL [41].

## Results

### Substrate selectivity of (MOUSE)NAT1*1 and (MOUSE)NAT2*1

**Figure 1:**
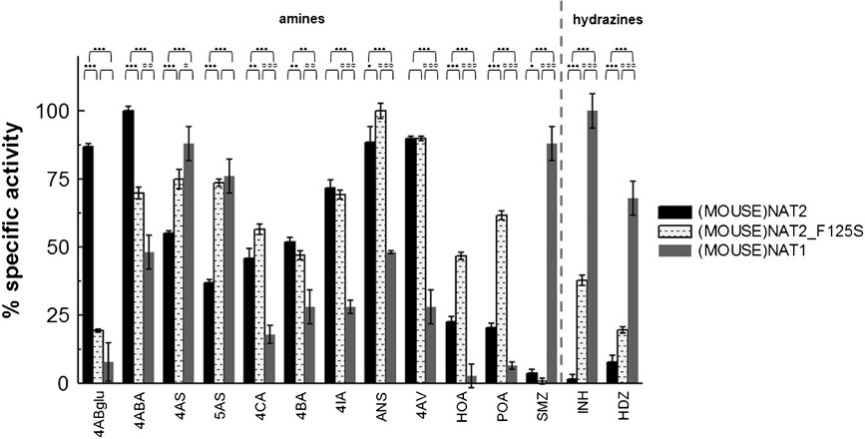
Substrate profiles of (MOUSE)NAT2*1, (MOUSE)NAT2_F125S and (MOUSE)NAT1*1. Substrate specific activity profiles of (MOUSE)NAT2*1 (black columns), (MOUSE)NAT2_F125S (dashed white columns) and (MOUSE)NAT1*1 (gray columns) are shown. Maximal specific activity values (normalised as 100% relative specific activity) were: 196 μM.sec^−1^.mg^−1^ for (MOUSE)NAT2*1 with 4ABA, 25 μM.sec^−1^.mg^−1^ for (MOUSE)NAT2_F125S with ANS and 104 μM.sec^−1^.mg^−1^ for (MOUSE)NAT1*1 with INH. A dashed line separates aromatic amines from hydrazines. The circles above the bars show the significance of the differences between (MOUSE)NAT2_F125S or (MOUSE)NAT1*1 and (MOUSE)NAT2*1 (●) and between (MOUSE)NAT2_F125S and (MOUSE)NAT1*1 (○) using ANOVA with Student’s t-test; ● or ○: *p* ≤0.05; ●● or ○○: *p* ≤0.01; ●●● or ○○○: *p* ≤0.001.

We expressed (MOUSE)NAT1*1 in *E. coli* Rosetta (DE3)pLysS and used the resulting recombinant protein to determine the activity of (MOUSE)NAT1*1 towards a panel of chemicals commonly used as NAT substrates (Additional file 3: Table S1). The highest specific activities were with the hydrazines INH and HDZ and the arylamine SMZ (Figure 1). A parallel screening experiment with (MOUSE)NAT2*1 yielded results corresponding to those published previously [22]. The differences between (MOUSE)NAT1*1 and (MOUSE)NAT2*1 for most substrates were statistically significant. (MOUSE)NAT1*1 did, however, have low but measurable activity towards certain arylamine substrates which are usually considered to be (MOUSE)NAT2-specific (4ABA and 4AS but not 4ABglu) and towards halogenated arylamines and alkyloxy- and aryloxy-substituted arylamines.

### Site-directed mutagenesis of (MOUSE)NAT2

Three (MOUSE)NAT2 mutants ((MOUSE)NAT2_F125S, (MOUSE)NAT2_R127G and (MOUSE)NAT2_R127L) were used to explore the effects of mutating a single residue within the active site on the substrate specificity of (MOUSE)NAT2. Two of these, (MOUSE)NAT2_R127G and (MOUSE)NAT2_R127L, were reported previously [8, 22]. The third, (MOUSE)NAT2_F125S was generated by site-directed mutagenesis, as described above and illustrated in Additional file 2: Figure S2.

The substrate panel listed in Additional file 3: Table S1 was used to characterise the activity profiles of the three mutants in comparison with that of (MOUSE)NAT2*1. The maximum specific activity of (MOUSE)NAT2_F125S (observed with EOA) was around 7.5 times lower than the maximal value of (MOUSE)NAT2*1 (observed with 4ABA), but the substrate preferences of (MOUSE)NAT2_F125S and (MOUSE)NAT2*1 were similar. Some discrepancies were, however, observed: the main differences observed were marked reductions in the relative rate of *N-*acetylation of the arylamines 4ABglu and 4ABA and an augmentation of activities towards the hydrazines INH and HDZ.

**Figure 2:**
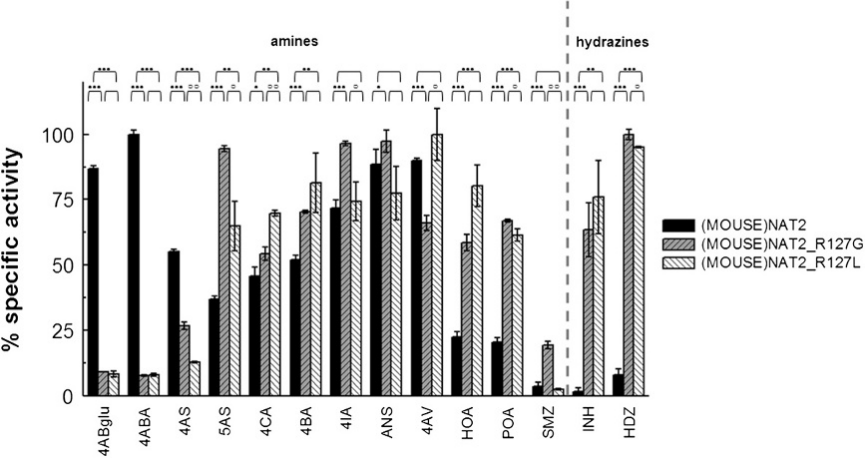
Substrate profiles of (MOUSE)NAT2*1, (MOUSE)NAT2_R127G and (MOUSE)NAT2_R127L. Substrate specific activity profiles of (MOUSE)NAT2*1 (black columns), (MOUSE)NAT2_R127G (dashed gray columns), (MOUSE)NAT2_R127L (dashed white columns) are shown. Maximal specific activity values (normalised as 100% relative specific activity) are: 196 μM.sec^−1^.mg^−1^ for (MOUSE)NAT2*1 with 4ABA, 1385 μM.sec^−1^.mg^−1^ for (MOUSE)NAT2_R127G with HDZ and 82.5 μM.sec^−1^.mg^−1^ for (MOUSE)NAT2_R127L with 4AV. A dashed line separates aromatic amines from hydrazines. The circles above the bars show the significance of the differences between (MOUSE)NAT2_R127G or (MOUSE)NAT2_R127L and (MOUSE)NAT2*1 (●) and between (MOUSE)NAT2_R127G and (MOUSE)NAT2_R127L (○) using ANOVA with Student’s t-test;● or ○: *p* ≤0.05; ●● or ○○: *p* ≤0.01; ●●● or ○○○: *p* ≤0.001.

Substituting R127 with glycine (R127G) or leucine (R127L) caused a dramatic loss of *N-*acetylation activity towards 4ABA and 4ABglu (Figure 2). Aminosalicylic acids were differentially *N-*acetylated by (MOUSE)NAT2*1 and its R127 mutants: (MOUSE)NAT2_R127G and (MOUSE)NAT2_R127L had significantly lower *N-*acetylation activity towards the (MOUSE)NAT2-specific substrate 4AS, but higher catalytic activity with 5AS compared with (MOUSE)NAT2*1. These mutations had no marked effects on the *N-*acetylation of halogenated arylamines and alkyloxy-substituted arylamines with small alkyl chains (ANS, 4AV), but those with extended alkyloxy or bulkier aryloxy substituents (hexyloxy in HOA and phenoxy in POA) were strongly preferred by both R127 mutants compared with (MOUSE)NAT2*1. The mutants also had much higher *N-*acetylation activity towards hydrazines such as HDZ.

Comparison of the substrate profiles of the two R127 mutants did not identify significant differences in their activity profiles. Overall, the best substrate for (MOUSE)NAT2_R127G was HDZ. The observed specific activity (1,385 μM.sec^−1^.mg^−1^) was 7 fold higher than the maximum specific activity of (MOUSE)NAT2*1 with 4ABA (196 μM.sec^−1^.mg^−1^), suggesting that (MOUSE)NAT2_R127G mutant had greater catalytic efficiency than (MOUSE)NAT2*1. In contrast, comparing the maximum specific activity value of (MOUSE)NAT2*1 (196 μM.sec^−1^.mg^−1^ with 4ABA) with that of (MOUSE)NAT2_R127L (82.5 μM.sec^−1^.mg^−1^ with 4AV) indicated that the maximum catalytic turnover of (MOUSE)NAT2_R127L was ~2.5 fold lower than that of (MOUSE)NAT2*1.

### Comparison of substrate selectivity among mammalian NATs

**Figure 3:**
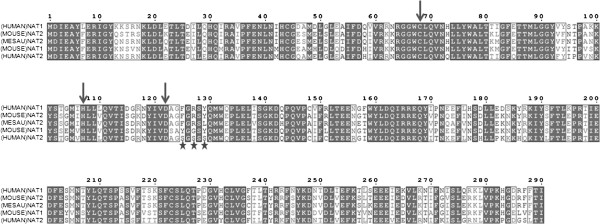
**Sequence alignment of five mammalian NAT proteins.** The primary sequences of human, mouse and Syrian hamster NAT proteins are aligned. Similar amino acids are highlighted by dark grey lettering in pale grey boxes; completely conserved residues are indicated by white lettering on a dark grey background. The residues of the catalytic triad are indicated by a blue arrow. Each residue putatively involved in substrate selectivity is indicated by a star. Alignments were generated using Clustal W [42] and the figure was prepared using ESPript 2.2 [43].

The assays described here were performed under the conditions described in our previous publications [8, 22]. We were therefore able to compare previously published substrate selectivity profiles of (HUMAN)NAT1*4, (HUMAN)NAT2*4 and (MESAU)NAT2*1 with those of (MOUSE)NAT1*1, (MOUSE)NAT2*1 and the three engineered (MOUSE)NAT2 mutants reported here (Table 1). In Table 1, the NAT enzymes are arranged according to divergence of the active site sequence from that of (HUMAN)NAT1*4 (Figure 3) [9] and plotted against arylamine and hydrazine substrates organised according to their chemical functionalities and physicochemical properties. Overall, as the active site diverged further from that of (HUMAN)NAT1*4, substrate preference moved from arylamines with negatively charged *para*-substituents towards aromatic amines and hydrazines without ionised, polar or *para*-substituents around the aromatic core. In particular, 4ABglu, 4ABA and 4AS have an acetyl acceptor amine with pK_aH_ <3 (Additional file 3: Table S1) and are subject to *N*-acetylation by (HUMAN)NAT1*4 and its homologues (MOUSE)NAT2*1 and (MESAU)NAT2*1. The other substrates have an acceptor amine with pK_aH_ ≥3. These are better substrates for (HUMAN)NAT2*4, (MOUSE)NAT1*1 and the two R127-mutated (MOUSE)NAT2 enzymes.

**Table 1:** **Arylamine activity profiles of native and engineered NATs in relation to substrate physicochemical properties**

			NAT enzymes: % specific activity values							
			(HUMAN) NAT1*4	(MOUSE) NAT2*1	(MESAU) NAT2*1	(MOUSE) NAT2 F125S	(MOUSE) NAT2 R127G	(MOUSE) NAT2 R127L	(MOUSE) NAT1*1	(HUMAN) NAT2*4
NAT substrates			F125	-	-	S	-	-	Y	S
			R127	-	-	-	G	L	G	S
			Y129	-	L	-	-	-	-	S
*Negatively charged arylamines at pH 8.0*	*Folate catabolites*	4AB glu	20 ± 1	87 ± 1	57 ± 3	19 ± 1	9 ± 1	8 ± 1	8 ± 7	3 ± 1
		4ABA	**100** ± 2	**100** ± 2	**100** ± 6	70 ± 2	8 ± 1	8 ± 1	48 ± 6	6 ± 1
	*Salicylic acids*	4AS	67 ± 2	55 ± 1	74 ± 3	75 ± 3	27 ± 1	13 ± 1	88 ± 6	7 ± 1
		5AS	64 ± 3	37 ± 1	30 ± 1	74 ± 1	94 ± 1	65 ± 9	76 ± 6	64 ± 2
*Neutral electron-rich arylamines at pH 8.0*	*Halogenated anilines*	4CA	36 ± 2	46 ± 4	61 ± 2	56 ± 2	54 ± 3	70 ± 1	18 ± 3	71 ± 3
		4BA	50 ± 1	52 ± 2	72 ± 1	47 ± 2	70 ± 1	81 ± 11	28 ± 6	72 ± 1
		4IA	63 ± 1	72 ± 3	81 ± 1	69 ± 2	**97** ± 1	74 ± 8	28 ± 3	65 ± 2
	*Alkyl- and aryl-oxyanilines*	ANS	56 ± 2	88 ± 6	89 ± 1	**100** ± 3	**97** ± 4	77 ± 10	48 ± 1	9 ± 2
		4AV	25 ± 2	90 ± 1	32 ± 1	90 ± 1	66 ± 3	**100** ± 10	28 ± 6	57 ± 3
		HOA	12 ± 4	23 ± 2	35 ± 1	47 ± 1	59 ± 3	80 ± 8	3 ± 4	51 ± 2
		POA	17 ± 7	20 ± 2	22 ± 1	62 ± 2	67 ± 1	61 ± 2	6 ± 1	62 ± 11
	*Other aniline*	SMZ	1 ± 1	4 ± 1	1 ± 1	1 ± 1	19 ± 2	3 ± 1	88 ± 6	46 ± 1
*Arylhydrazines*		INH	0 ± 2	2 ± 2	1 ± 1	38 ± 2	63 ± 10	76 ± 14	**100** ± 6	62 ± 2
		HDZ	2 ± 1	8 ± 2	3 ± 1	19 ± 1	**100** ± 2	**95** ± 1	68 ± 6	**100** ± 1

Percentage specific activity values of native and engineered mammalian NAT proteins are shown. A value of 100% is attributed to the substrate towards which the isoform in question has the highest specific activity. All results shown are the mean of 3 measurements ± standard deviation. Percentages ≥90% for each isoenzyme were compared by ANOVA using a Student’s t-test and statistically similar values are emboldened. Isoforms are ordered according to increasing number of residue differences within the active site in relation to (HUMAN)NAT1*4. Residues identical to those in (HUMAN)NAT1*4 are labelled with a hyphen; residues different from the corresponding residue of (HUMAN)NAT1*4 are specified. Substrates are ordered according to the number of negative charges at pH 8.0, then to electron-richness and increasing size of aromatic substituents. The chemical structures and physicochemical properties of the chemicals used as substrates for NAT in this study are shown in Additional file 3: Table S1.

In general, the results obtained corresponded with previous reports of functional similarities between (HUMAN)NAT1*4, (MOUSE)NAT2*1 and (MESAU)NAT2*1 [8, 22]; however, the substrate profile of (MOUSE)NAT1*1 did not, as expected, correspond to that of (HUMAN)NAT2*4 [29, 31]. Indeed, (MOUSE)NAT1*1 was able to *N-*acetylate substrates characteristic of both (HUMAN)NAT1*4 (4ABA and 4AS) and (HUMAN)NAT2*4 (SMZ, INH and HDZ).

### Inhibition studies

The inhibitory potency and colorimetric properties of the (HUMAN)NAT1-selective inhibitor naphthoquinone **1** were also explored in relation to alterations at positions 125, 127 and 129 using (MOUSE)NAT2_F125S, (MOUSE)NAT1*1 and (HUMAN)NAT2*4, comparing the results with those of previous studies using (HUMAN)NAT1*4, (MESAU)NAT2*1, (MOUSE)NAT2*1, (MOUSE)NAT2_R127G and (MOUSE)NAT2_R127L [24–26] (Table 2). The lowest IC_50_ values for naphthoquinone **1** were exhibited by (HUMAN)NAT1*4 and (MOUSE)NAT2*1 enzymes, both of which possess the triad F125, R127 and Y129. Naphthoquinone **1** was at least an order of magnitude more potent against (HUMAN)NAT1*4 and (MOUSE)NAT2*1 (IC_50_ values of 5.3 and 1.3 μM, respectively) than the other NATs used in this study. When pure recombinant (MOUSE)NAT2_F125S was incubated with naphthoquinone **1** under the conditions reported previously, it did not shift the λ_max_ of naphthoquinone **1** towards longer wavelengths (585–625 nm), in contrast to previous observations with (HUMAN)NAT1*4 and (MOUSE)NAT2*1 (Additional file 4: Figure S3, Table 2).

**Table 2:** **Effects of naphthoquinone 1 on the activity of mammalian NATs and their colorimetric detection**

Enzyme		(HUMAN) NAT1*4	(MOUSE) NAT2*1	(MESAU) NAT2*1	(MOUSE) NAT2_F125S	(MOUSE) NAT2_R127G	(MOUSE) NAT2_R127L	(MOUSE) NAT1*1	(HUMAN) NAT2*4
Primary sequence identity *versus* (HUMAN)NAT1*4		100	82	81	82	82	82	74	71
Active site residues	125	F	F	F	**S**	F	F	**Y**	**S**
	127	R	R	R	R	**G**	**L**	**G**	**S**
	129	Y	Y	**L**	Y	Y	Y	Y	**S**
IC_50_ with naphthoquinone **1** (*μ*M)		5.3^a^	1.3^a,b^	89.0^c^	68.7	51.7^a^	102.5^a^	129.7	>100
*λ*_max_ of naphthoquinone **1** (nm)		585^a,b^	625^a,b^	525^c^	498	498^a^	498^a^	498^b^	498^a^

IC_50_ values were calculated using decreasing concentrations of naphthoquinone 1. NAT activity was measured following the hydrolysis rate of AcCoA (400 μM) in the presence of 4ABA for (HUMAN)NAT1*4, (MOUSE)NAT2*1 and (MESAU)NAT2*1; 5AS for (MOUSE)NAT2 mutants and (MOUSE)NAT1*1; and 2-aminofluorene for (HUMAN)NAT2*4. ^a^adapted from [25] ^b^adapted from [24] ^c^adapted from [26]. Residues which are different from the corresponding residues in (HUMAN)NAT1*4 are labeled in bold.

### *In silico*analysis of substrate selectivity in mammalian NATs

The structural features underlying substrate selectivity were investigated by *in silico* analysis of interactions between the NAT proteins and their arylamine substrates.

Structural models of (MOUSE)NAT2*1 and (MESAU)NAT2*1 were generated using the crystal structure of (HUMAN)NAT1*4 [PDB:2PQT] [9] because these three isoforms have >80% amino acid identity (Additional file 5: Table S2). Swiss-Model simulations generated models of (MOUSE)NAT2*1 and (MESAU)NAT2*1 which were of high quality according to their scoring functions and background noise, thus confirming the reliability of the template used. The mutations (MOUSE)NAT2_F125S, (MOUSE)NAT2_R127G and (MOUSE)NAT2_R127L did not abolish the catalytic reactivity compared with the reference enzyme, so each individual residue modification was considered unlikely to have altered overall protein folding. The same modelling procedure was therefore used to create structural models of (MOUSE)NAT2_F125S, (MOUSE)NAT2_R127G and (MOUSE)NAT2_R127L. However, attempts to model (MOUSE)NAT1*1 (64% identity; Additional file 5: Table S2) based on the structure of (HUMAN)NAT2*4 [PDB:2PFR] [9] did not yield reliable results.

**Figure 4:**
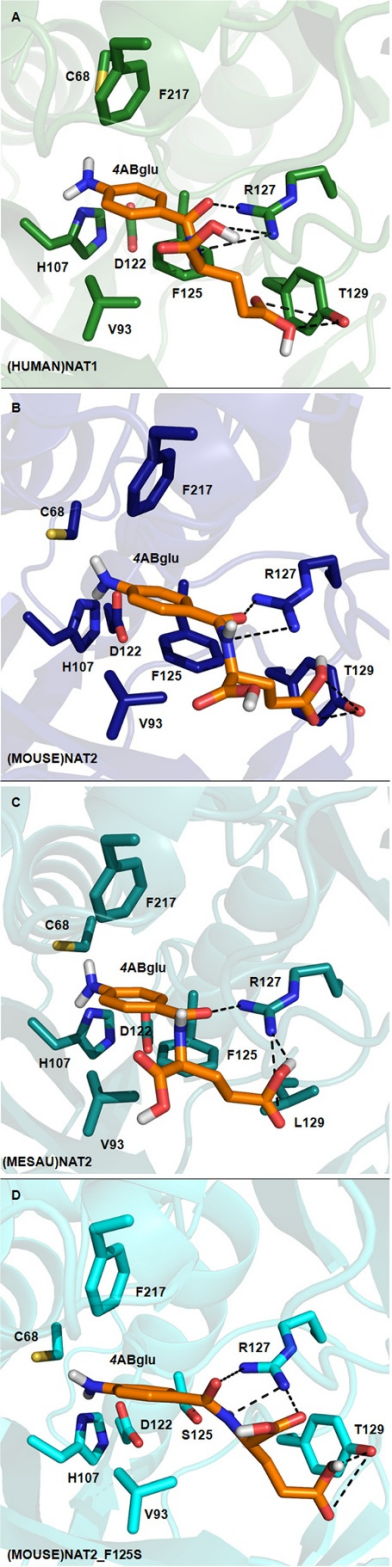
Substrate binding pockets of (HUMAN)NAT1*4, (MOUSE)NAT2*1, (MESAU)NAT2*1 and (MOUSE)NAT2_F125S with 4ABglu docked. Maximised view of the active sites of **(A)**: (HUMAN)NAT1*4; **(B)**: (MOUSE)NAT2*1; **(C)**: (MESAU)NAT2*1; **(D)** (MOUSE)NAT2_F125S with the arylamine substrate 4ABglu docked. The overall structure of each NAT enzyme is drawn as a ribbon diagram: (HUMAN)NAT1*4 is coloured in green [PDB:2PQT] [9] (MOUSE)NAT2*1 model in dark blue, (MESAU)NAT2*1 model in cyan and (MOUSE)NAT2_F125S model in pale blue. The side chains of the key residues involved in substrate binding within the active site are drawn in stick representation and labelled with carbon atoms in the corresponding colour of the enzyme, nitrogen in blue, oxygen in red and sulphur in yellow. The arylamine substrate 4ABglu is labelled with carbon atoms in orange, nitrogen in blue, oxygen in red and polar hydrogen in gray. The figures were generated using PyMOL [41].

Substrates were docked into the catalytic site of each enzyme as shown in the selectivity profile summarised in Table 1. 4ABglu was docked in the active sites of (HUMAN)NAT1*4, (MOUSE)NAT2*1, (MESAU)NAT2*1 and (MOUSE)NAT2_F125S (Figure 4). The affinity energies of all these simulations were low (−8.0/-7.0 kcal.mol^−1^) and probable polar and hydrophobic interactions were revealed within the enzyme-substrate complexes. For example, the amide functionality of 4ABglu could point towards the guanidinium of R127 *via* hydrogen bonds (<3.2 Å); the Cγ carboxylic group of glutamate could form a hydrogen bridge with the hydroxyl tail of Y129 (<3.1 Å) in (HUMAN)NAT1*4, (MOUSE)NAT2*1 and (MOUSE)NAT2_F125S; and/or the aromatic substrate core could interact *via* π-π stacking (<3.8 Å) with the hydrophobic plane defined by the isopropyl moiety of valine 93 (V93) and the phenyl ring of F125 in all the reference enzymes. The results obtained with 4ABA support the postulated interactions inferred from 4ABglu, suggesting a polar interaction between the carboxylate of 4ABA and the guanidinium of R127 at pH 8.0 (~3.6 Å) and hydrophobic stacking of the aromatic portion of 4ABA on the apolar flat surface defined by the side chains of V93 and F125 (~4 Å) (Additional file 6: Figure S4).

**Figure 5:**
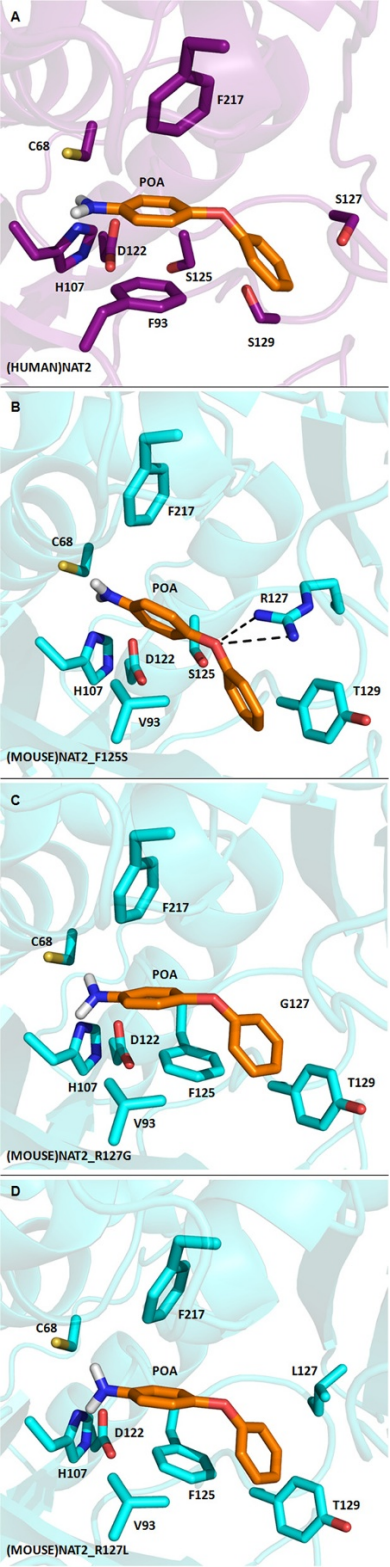
Substrate binding pockets of (HUMAN)NAT2*4, (MOUSE)NAT2_F125S, (MOUSE)NAT2_R127G and (MOUSE)NAT2_R127L with POA docked. Maximised view of the active sites of **(A)**: (HUMAN)NAT2*4; **(B)**: (MOUSE)NAT2_F125S; **(C)**: (MOUSE)NAT2_R127G; **(D)**: (MOUSE)NAT2_R127L with the arylamine substrate POA docked. The overall structure of each NAT enzyme is drawn as a ribbon diagram: (HUMAN)NAT2*4 is coloured in mauve [PDB:2PFR] [9] and (MOUSE)NAT2 mutants in light blue. The side chains of the key residues involved in substrate binding within the active site are drawn in stick representation and labelled with carbon atoms in the corresponding colour of the enzyme, nitrogen in blue, oxygen in red and sulphur in yellow. The arylamine substrate POA is labelled with carbon atoms in orange, nitrogen in blue, oxygen in red and polar hydrogen in gray. The figures were generated using PyMOL [41].

We also attempted to model the selective hydrazine substrates INH and HDZ within the active site of (HUMAN)NAT2*4 and the (MOUSE)NAT2 mutants. No proximity between the hydrazine functionality and C68 thiolate compatible with the NAT catalytic mechanism [44] was observed, possibly because of the smaller steric size of the substrate docked. However, when a bulkier selective arylamine substrate such as POA was docked into (HUMAN)NAT2*4 and the three (MOUSE)NAT2 mutants, the results indicated proximity between the primary amine of the substrate and the key active site residues C68 and H106 (<4 Å) (Figure 5). These simulations indicated that the 4-aminoarene of POA could be sandwiched between the benzyl ring of F217 and the apolar side chain of F93 in (HUMAN)NAT2*4 and F125 in (MOUSE)NAT2_R127G and (MOUSE)NAT2_R127L *via* hydrophobic interactions. In the (MOUSE)NAT2 mutants, the phenoxy group of POA was predicted to make further π-stacking interactions with the side chain of Y129 (~3.9 Å), whereas the equivalent residue in (HUMAN)NAT2*4, S129, is incapable of this interaction.

## Discussion

The present study has extended our understanding of (MOUSE)NAT1 by demonstrating that it is functionally different from (HUMAN)NAT2. In addition to its known activity in *N*-acetylating the hydrazine INH and the arylamine SMZ [29, 30], (MOUSE)NAT1*1 was shown to have significant activity towards the hydrazine HDZ and two arylamine substrates which were previously considered to be (HUMAN)NAT1 and (MOUSE)NAT2-specific (4ABA and 4AS).

The substrate-binding interactions involved in *N*-acetylation were previously characterised using nuclear magnetic resonance [33, 45], demonstrating that an arylamine or hydrazine could bind to a non-acetylated NAT active site. In the present study, arylamine substrates were docked within the catalytic pockets of different non-acetylated NATs *in silico* in order to identify possible selective interactions underlying the formation of the enzyme-substrate complex.

In (HUMAN)NAT1*4, the positively charged guanidinium (at pH 8.0) of R127 plays an essential role in recognising the negatively charged carboxylate of 4ABA, as illustrated by modelling 4ABA (Additional file 6: Figure S4) or 4AS [9] within the active site. The significant *N*-acetylation activity of (MOUSE)NAT1*1 towards 4ABA, 4AS and ANS in the present study was difficult to reconcile with the presence of an apolar G127 instead of an R127. However, it is possible that the presence of a sterically smaller residue (G) at this position in (MOUSE)NAT1*1 permits bulky or highly polar substrates to enter and allows their charged *para*-substituents to interact with other polar side chains in the active site (e.g. Y125 and Y129). Similarly, essential π-π stacking interactions observed between the aromatic core of 4ABA and F125, as seen in (HUMAN)NAT1*4, may be permitted by the side chain arene of Y125 within the active site of (MOUSE)NAT1*1. The lack of a structural model suitable for docking studies and the restricted commonality of substrate selectivity between (MOUSE)NAT1*1 and either of the two human NATs precluded the construction of a structural model for (MOUSE)NAT1*1, so this hypothesis could not be tested.

When 4ABglu was docked into the structure of (HUMAN)NAT1*4 and models of (MOUSE)NAT2*1 and (MESAU)NAT2*1, the essential role of R127 in forming ionic interactions with the electron-negative *para*-substituent of the substrate was evident; this may explain why a bulky charged arylamine such as 4ABglu can enter a small, very hydrophobic microenvironment such as the active site crevice of (HUMAN)NAT1*4 [9]. The preference of (HUMAN)NAT1*4 and its homologues (MOUSE)NAT2*1 and (MESAU)NAT2*1 for arylamine substrates with a negatively charged *para*-substituent (e.g. 4ABA and 4AS) may therefore be due to the positively charged guanidinium moiety of R127.

Previous studies have shown that mutation of F125 to S125 modifies the catalytic preference of (HUMAN)NAT1*4 from the conventional probe substrate 4AS to SMZ [27]. When we examined the effect of the equivalent mutation on (MOUSE)NAT2, no such shift in substrate preference was observed; neither did the general substrate preferences of (MOUSE)NAT2 change to resemble those of (MOUSE)NAT1*1. The higher reactivity of (MOUSE)NAT2_F125S with conformationally flexible hydrazines than with planar arylamines could be associated with increased active site space in (MOUSE)NAT2*1 after the substitution of the bulky benzyl group of F with the less hindering hydroxymethyl of S.

Site-directed mutagenesis of R127 to G127 or L127 within (MOUSE)NAT2 markedly altered the enzyme’s substrate selectivity. Overall, the non-polar side chains of G and L appeared to have similar effects in terms of modifying the substrate preferences of (MOUSE)NAT2: the metabolism of 4ABglu and 4ABA was dramatically decreased whereas *N*-acetylation of the hydrazines INH and HDZ, which are commonly used as probe substrates for (MOUSE)NAT1 and (HUMAN)NAT2, was augmented. R127 seemed to play a crucial role in discriminating between arylamines with highly hydrophilic substituents and hydrazines with less polar aromatic functional groups. Moreover, the (MOUSE)NAT2_R127G mutation improved the overall catalytic activity of the enzyme, possibly due to the larger size of the active site cavity generated after substitution. Similarly, when the arylamine POA was docked into the active sites of (MOUSE)NAT2_F125S, (MOUSE)NAT2_R127G and (MOUSE)NAT2_R127L, the results suggested that accommodation of this substrate *via* stacking interactions is facilitated by the larger cavity created by these single substitutions. When POA was docked into the active pocket of (HUMAN)NAT2*4, the results of substrate fitting were very similar to previous modelling results obtained with SMZ [9], consistent with the preference of (HUMAN)NAT2*4 for apolar and flexible substrates [11].

The individual (MOUSE)NAT2 mutants tested did not affect the specificity of (MOUSE)NAT2 for SMZ, which is a very poor substrate for this isoform. This may be related to the need for a larger active site to accommodate SMZ, which is a bulky arylamine; a single amino acid change may not be sufficient to permit access to the active site. Further 3D structural data and thermal stability studies on the mutants would help to ascertain the extent of the folding perturbations produced by each mutation. However, it is also intriguing that small hydrazines such as INH and phenylhydrazine are poor substrates for (HUMAN)NAT1 and its rodent homologues [8, 22], whereas arylamines of similar steric size such as 4ABA, 4AS and ANS are good substrates.

While the amine nitrogen and the aromatic carbons of an arylamine substrate are on the same plane, crystallographic studies of INH, HDZ and phenylhydrazine as single molecules [46–50] and protein co-crystallised ligands [51, 52], showed that the hydrazine bond could also be off the plane defined by the remaining aryl and acyl carbon atoms, thereby giving the hydrazine substrate a non-planar conformation. Our docking simulations using the crystal structure of (HUMAN)NAT1*4 and the model of (MOUSE)NAT2*1 show that the bulky side chain of R127, the benzyl ring of F125 and the 4-hydroxybenzyl of Y129 create a characteristic constrained microenvironment which appears to allow preferable accommodation of planar arylamines rather than conformationally flexible hydrazines in the active site of (HUMAN)NAT1*4. This is consistent with the volumes of the active sites in human NAT structures: the (HUMAN)NAT1*4 cavity has a volume of 162 Å^3^, whereas (HUMAN)NAT2*4 (with S at positions 125, 127 and 129) has a larger active pocket (257 Å^3^). In this context it is also noteworthy that the crystal structures of prokaryotic NATs from *Mycobacterium tuberculosis, M. marinum* and *M. smegmatis*, which preferentially *N*-acetylate conformationally flexible hydrazine substrates, have much larger active sites (>200 Å^3^) than (HUMAN)NAT1*4 [53–55].

Recent studies have suggested that F125, R127 and Y129 in the active sites of (HUMAN)NAT1*4 and (MOUSE)NAT2*1 are essential for selective recognition and binding of the potent inhibitor naphthoquinone **1** [24–26]. In particular, ionic recognition between the conjugate base of naphthoquinone **1** and the guanidinium moiety of R127 is known to be essential for inhibitor binding. The results shown here confirm that these key residues are required for the interaction with naphthoquinone **1**, since the absence of any of them results in a protein which is resistant to inhibition and colour shifting in response to naphthoquinone **1** (Table 2).

Overall, our results suggest that the substrate selectivity of the mammalian NATs investigated in this study is influenced by two major factors; firstly, the planarity of the acceptor arylamine and the polarity of its *para* substituent (which appear to affect both the type of the intermolecular interactions within the enzyme and the pK_aH_ strength and nucleophilicity of the acceptor amine); and secondly, the size of the catalytic cavity and its overall polarity, as determined by the residues at positions 125, 127 and 129. In particular, F125 in (HUMAN)NAT1*4 and (MOUSE)NAT2*1 appears to play a key role in discriminating between planar arylamines and conformationally flexible hydrazines, while the ionised (at physiological pH) side chain of R127 perturbs the characteristic hydrophobicity of the NAT active site and, in cooperation with the 4-hydroxybenzyl side chain of Y129, contributes to the narrowness of the (HUMAN)NAT1*4 active site.

## Conclusions

In conclusion, this evaluation of the substrate profiles of various native and engineered mammalian NATs in the context of their structures has highlighted the features which influence NAT substrate selectivity in mammals. The virtual models of mammalian NATs generated in this study, used in conjunction with X-ray structures of human NATs, constitute a rich resource for investigating the roles of particular residues within the NAT active site in relation to both NAT activity and inhibitor selectivity. Three non-catalytic residues within (HUMAN)NAT1*4 (F125, R127 and Y129) contribute both to substrate recognition and inhibitor binding by participating in distinctive intermolecular interactions and maintaining the steric conformation of the catalytic pocket. These active site residues contribute to the definition of substrate and inhibitor selectivity, an understanding of which is essential for facilitating the design of second generation (HUMAN)NAT1-selective inhibitors for diagnostic, prognostic and therapeutic purposes. In particular, since the expression of (HUMAN)NAT1 is related to the development and progression of oestrogen-receptor-positive breast cancer, these structure-based tools will facilitate the ongoing design of candidate compounds for use in (HUMAN)NAT1-positive breast tumours.

### Chemical compounds studied in this article

Aminobenzoyl glutamate (PubChem CID: 5103842), 4-aminobenzoic acid (PubChem CID: 978), 4-aminosalicylate (PubChem CID: 4649), 5-aminosalicylate (PubChem CID: 4075), 4-chloroaniline (PubChem CID: 7812), 4-methoxyaniline (PubChem CID: 7732), 4-phenoxyaniline (PubChem CID: 8764), hydralazine (PubChem CID: 3637), isoniazid (PubChem CID: 3767), sulfamethazine (PubChem CID: 5327).

## Electronic supplementary material

Additional file 1: Figure S1: Selective functionality of (HUMAN)NAT1*4 and (MOUSE)NAT2*1. Two catalytic reactions are selective to (HUMAN)NAT1*4 and (MOUSE)NAT2*1 amongst mammalian NATs: the *N*-acetylation of the folate catabolite 4ABglu and the folate-dependent hydrolysis of AcCoA. Both reactions are selectively inhibited by naphthoquinone **1** (shown in red). (TIFF 55 KB)

Additional file 2: Figure S2: Multiple sequence alignment of (MOUSE)Nat2*1 gene and *(MOUSE)Nat2_F125S* gene. Alignment of (MOUSE)Nat2*1 sequence and the forward and reverse sequences obtained after mutagenesis was conducted by ClustalW [42]. The single mutated nucleotide is highlighted in red. *indicates nucleotide identity among all three genetic sequences. No additional mutations were generated during the process of site directed mutagenesis. (TIFF 130 KB)

Additional file 3: Table S1: Chemical NAT substrates used in this study. Ionic charges of substrates at assay pH 8.0 are shown according to their pK_a(H)_ values [56–61]. (DOCX 63 KB)

Additional file 4: Figure S3: Visible spectra of naphthoquinone **1** in the presence of different mammalian NAT variants. Naphthoquinone **1** (15 μM) was incubated with 20 mM Tris–HCl, pH 8.0, 5% DMSO (v/v) (red line) or NAT variants (30 μM): (MOUSE)NAT2*1 (blue line); (MOUSE)NAT2_F125S (yellow line)). Wavelength scans from 800 to 350 nm were recorded against the appropriate blank (20 mM Tris–HCl, pH 8.0, 5% DMSO (v/v)). (TIFF 71 KB)

Additional file 5: Table S2: Comparison of eukaryotic NAT sequence identity and similarity. Percentage identity (no shade) and similarity (grey shade) values were calculated amongst five mammalian NATs using BLAST2 sequences. (DOCX 12 KB)

Additional file 6: Figure S4: Substrate binding pockets of (HUMAN)NAT1*4 with 4ABA docked. Maximised view of the active sites of (HUMAN)NAT1*4 with the arylamine substrate 4ABA docked. The overall structure of (HUMAN)NAT1*4 is drawn in ribbon diagram (green) [PDB:2PQT]. The side chain of the key residues involved in substrate binding within the active site are drawn in stick representation and labelled with carbon atoms in the colour of the enzyme, nitrogen in blue, oxygen in red and sulphur in yellow. The arylamine substrate 4ABA is labelled with carbon atoms in orange, nitrogen in blue, oxygen in red and polar hydrogen in gray. The figures were generated using PyMOL [41]. (TIFF 421 KB)

## Abbreviations

4ABA: 4-aminobenzoic acid
4ABglu: 4-aminobenzoylglutamate
4AS: 4-aminosalicylate
5AS: 5-aminosalicylate
4AV: 4-aminoveratrole
4BA: 4-bromoaniline
4CA: 4-chloroaniline
4IA: 4-iodoaniline
ANS: 4-methoxyaniline
AcCoA: Acetyl coenzyme A
DMSO: Dimethyl sulphoxide
HOA: 4-hexyloxyaniline
HDZ: Hydralazine
INH: Isoniazid
NAT: Arylamine *N-*acetyltransferase
POA: 4-phenoxyaniline
SMZ: Sulfamethazine.

## References

[1] Weber WW, Hein DW (1985). *N-*acetylation pharmacogenetics. Pharmacol Rev.

[2] Sim E, Abuhammad A, Ryan A (2014). Arylamine *N-*acetyltransferases: from drug metabolism and pharmacogenetics to drug discovery. Br J Pharmacol.

[3] Blum M, Grant DM, McBride W, Heim M, Meyer UA (1990). Human arylamine *N-*acetyltransferase genes: isolation, chromosomal localization, and functional expression. DNA Cell Biol.

[4] Hickman D, Risch A, Buckle V, Spurr NK, Jeremiah SJ, McCarthy A, Sim E (1994). Chromosomal localization of human genes for arylamine *N-*acetyltransferase. Biochem J.

[5] Hein DW (2009). *N-*acetyltransferase SNPs: emerging concepts serve as a paradigm for understanding complexities of personalized medicine. Expert Opin Drug Metab Toxicol.

[6] Hein DW, Doll MA, Fretland AJ, Leff MA, Webb SJ, Xiao GH, Devanaboyina US, Nangju NA, Feng Y (2000). Molecular genetics and epidemiology of the NAT1 and NAT2 acetylation polymorphisms. Cancer Epidemiol Biomarkers Prev.

[7] Ohsako S, Deguchi T (1990). Cloning and expression of cDNAs for polymorphic and monomorphic arylamine *N-*acetyltransferases from human liver. J Biol Chem.

[8] Kawamura A, Graham J, Mushtaq A, Tsiftsoglou SA, Vath GM, Hanna PE, Wagner CR, Sim E (2005). Eukaryotic arylamine *N-*acetyltransferase: investigation of substrate specificity by high-throughput screening. Biochem Pharmacol.

[9] Wu H, Dombrovsky L, Tempel W, Martin F, Loppnau P, Goodfellow GH, Grant DM, Plotnikov AN (2007). Structural basis of substrate-binding specificity of human arylamine *N-*acetyltransferases. J Biol Chem.

[10] Sim E, Lack N, Wang CJ, Long H, Westwood I, Fullam E, Kawamura A (2008). Arylamine *N-*acetyltransferases: structural and functional implications of polymorphisms. Toxicology.

[11] Zhou X, Ma Z, Dong D, Wu B (2013). Arylamine *N-*acetyltransferases: a structural perspective. Br J Pharmacol.

[12] Butcher NJ, Arulpragasam A, Pope C, Minchin RF (2003). Identification of a minimal promoter sequence for the human *N-*acetyltransferase type I gene that binds AP-1 (activator protein 1) and YY-1 (Yin and Yang 1). Biochem J.

[13] Minchin RF, Hanna PE, Dupret JM, Wagner CR, Rodrigues-Lima F, Butcher NJ (2007). Arylamine *N-*acetyltransferase I. Int J Biochem Cell Biol.

[14] Sim E, Walters K, Boukouvala S (2008). Arylamine *N-*acetyltransferases: from structure to function. Drug Metab Rev.

[15] Boukouvala S, Fakis G (2005). Arylamine *N-*acetyltransferases: what we learn from genes and genomes. Drug Metab Rev.

[16] Minchin RF (1995). Acetylation of *p-*aminobenzoylglutamate, a folic acid catabolite, by recombinant human arylamine *N-*acetyltransferase and U937 cells. Biochem J.

[17] Ward A, Summers MJ, Sim E (1995). Purification of recombinant human *N-*acetyltransferase type 1 (NAT1) expressed in *E. coli* and characterization of its potential role in folate metabolism. Biochem Pharmacol.

[18] Laurieri N, Dairou J, Egleton JE, Stanley LA, Russell AJ, Dupret JM, Sim E, Rodrigues-Lima F (2014). From arylamine *N-*Acetyltransferase to folate-dependent acetyl CoA hydrolase: impact of folic acid on the activity of (HUMAN)NAT1 and its homologue (MOUSE)NAT2. PLoS One.

[19] Sugamori KS, Brenneman D, Wong S, Gaedigk A, Yu V, Abramovici H, Rozmahel R, Grant DM (2007). Effect of arylamine acetyltransferase *Nat3* gene knockout on *N-*acetylation in the mouse. Drug Metab Dispos.

[20] Boukouvala S, Price N, Sim E (2002). Identification and functional characterization of novel polymorphisms associated with the genes for arylamine *N-*acetyltransferases in mice. Pharmacogenetics.

[21] Fretland AJ, Doll MA, Gray K, Feng Y, Hein DW (1997). Cloning, sequencing, and recombinant expression of NAT1, NAT2, and NAT3 derived from the C3H/HeJ (rapid) and A/HeJ (slow) acetylator inbred mouse: functional characterization of the activation and deactivation of aromatic amine carcinogens. Toxicol Appl Pharmacol.

[22] Kawamura A, Westwood I, Wakefield L, Long H, Zhang N, Walters K, Redfield C, Sim E (2008). Mouse *N-*acetyltransferase type 2, the homologue of human *N-*acetyltransferase type 1. Biochem Pharmacol.

[23] Stanley LA, Mills IG, Sim E (1997). Localization of polymorphic *N-*acetyltransferase (NAT2) in tissues of inbred mice. Pharmacogenetics.

[24] Laurieri N, Crawford MH, Kawamura A, Westwood IM, Robinson J, Fletcher AM, Davies SG, Sim E, Russell AJ (2010). Small molecule colorimetric probes for specific detection of human arylamine *N-*acetyltransferase 1, a potential breast cancer biomarker. J Am Chem Soc.

[25] Laurieri N, Egleton JE, Varney A, Thinnes CC, Quevedo CE, Seden PT, Thompson S, Rodrigues-Lima F, Dairou J, Dupret JM, Russell AJ, Sim E (2013). A novel color change mechanism for breast cancer biomarker detection: naphthoquinones as specific ligands of human arylamine *N-*acetyltransferase 1. PLoS One.

[26] Egleton JE, Thinnes CC, Seden PT, Laurieri N, Lee SP, Hadavizadeh KS, Measures AR, Jones AM, Thompson S, Varney A, Wynne GM, Ryan A, Sim E, Russell AJ (2014). Structure–activity relationships and colorimetric properties of specific probes for the putative cancer biomarker human arylamine *N-*acetyltransferase 1. Bioorg Med Chem.

[27] Goodfellow GH, Dupret JM, Grant DM (2000). Identification of amino acids imparting acceptor substrate selectivity to human arylamine acetyltransferases NAT1 and NAT2. Biochem J.

[28] Dupret JM, Goodfellow GH, Janezic SA, Grant DM (1994). Structure-function studies of human arylamine *N-*acetyltransferases NAT1 and NAT2. Functional analysis of recombinant NAT1/NAT2 chimeras expressed in *Escherichia coli*. J Biol Chem.

[29] Estrada-Rodgers L, Levy GN, Weber WW (1998). Substrate selectivity of mouse *N-*acetyltransferases 1, 2, and 3 expressed in COS-1 cells. Drug Metab Dispos.

[30] Martell KJ, Levy GN, Weber WW (1992). Cloned mouse *N-*acetyltransferases: enzymatic properties of expressed *Nat-1* and *Nat-2* gene products. Mol Pharmacol.

[31] Sugamori KS, Wong S, Gaedigk A, Yu V, Abramovici H, Rozmahel R, Grant DM (2003). Generation and functional characterization of arylamine *N-*acetyltransferase *Nat1/Nat2* double-knockout mice. Mol Pharmacol.

[32] Kelly SL, Sim E (1994). Arylamine *N-*acetyltransferase in Balb/c mice: identification of a novel mouse isoenzyme by cloning and expression *in vitro*. Biochem J.

[33] Zhang N, Liu L, Liu F, Wagner CR, Hanna PE, Walters KJ (2006). NMR-based model reveals the structural determinants of mammalian arylamine *N-*acetyltransferase substrate specificity. J Mol Biol.

[34] Butcher NJ, Minchin RF (2012). Arylamine *N-*acetyltransferase 1: a novel drug target in cancer development. Pharmacol Rev.

[35] Andres HH, Klem AJ, Szabo SM, Weber WW (1985). New spectrophotometric and radiochemical assays for acetyl-CoA: arylamine *N-*acetyltransferase applicable to a variety of arylamines. Anal Biochem.

[36] Brooke EW, Davies SG, Mulvaney AW, Pompeo F, Sim E, Vickers RJ (2003). An approach to identifying novel substrates of bacterial arylamine *N-*acetyltransferases. Bioorg Med Chem.

[37] Arnold K, Bordoli L, Kopp J, Schwede T (2006). The SWISS-MODEL workspace: a web-based environment for protein structure homology modelling. Bioinformatics.

[38] Kiefer F, Arnold K, Kunzli M, Bordoli L, Schwede T (2009). The SWISS-MODEL repository and associated resources. Nucleic Acids Res.

[39] Peitsch MC (1995). Protein modeling by E-Mail. Bio-Technol.

[40] Trott O, Olson AJ (2010). AutoDock Vina: improving the speed and accuracy of docking with a new scoring function, efficient optimization, and multithreading. J Comput Chem.

[41] DeLano WL (2002). PyMOL: An open-source molecular graphics tool. DeLano Scientific, San Carlos, California, USA.

[42] Thompson JD, Higgins DG, Gibson TJ (1994). CLUSTAL W: improving the sensitivity of progressive multiple sequence alignment through sequence weighting, position-specific gap penalties and weight matrix choice. Nucleic Acids Res.

[43] Gouet P, Courcelle E, Stuart DI, Metoz F (1999). ESPript: analysis of multiple sequence alignments in PostScript. Bioinformatics.

[44] Westwood IM, Kawamura A, Fullam E, Russell AJ, Davies SG, Sim E (2006). Structure and mechanism of arylamine *N-*acetyltransferases. Curr Top Med Chem.

[45] Delgoda R, Lian LY, Sandy J, Sim E (2003). NMR investigation of the catalytic mechanism of arylamine *N-*acetyltransferase from *Salmonella typhimurium*. Biochim Biophys Acta.

[46] Abboud KA, Smith DW, Wagener KB (1993). Structure of cyclo-1,1′,4,4′-bis(1,1,3,3-tetramethyl-1,3-disiloxanediyl)dibenzene. Acta Crystallogr C.

[47] Borba A, Gomez-Zavaglia A, Fausto R (2009). Molecular structure, infrared spectra, and photochemistry of isoniazid under cryogenic conditions. J Phys Chem A.

[48] Srinivasan S, Swaminathan S (1968). The crystal structure of phenyl hydrazine, C6H5 · NH · NH2. Zeitschrift für Kristallographie.

[49] Okabe N, Fukuda H, Nakamura T (1993). Structure of hydralazine hydrochloride. Acta Crystallogr C.

[50] Lemmerer A (2012). Covalent assistance to supramolecular synthesis: modifying the drug functionality of the antituberculosis API isoniazid *in situ* during co-crystallization with GRAS and API compounds. Cryst Eng Comm.

[51] Abuhammad AM, Lowe ED, Fullam E, Noble M, Garman EF, Sim E (2010). Probing the architecture of the *Mycobacterium marinum* arylamine *N-*acetyltransferase active site. Protein Cell.

[52] Sandy J, Mushtaq A, Kawamura A, Sinclair J, Sim E, Noble M (2002). The structure of arylamine *N*-acetyltransferase from *Mycobacterium smegmatis*–an enzyme which inactivates the anti-tubercular drug, isoniazid. J Mol Biol.

[53] Sinclair JC, Sandy J, Delgoda R, Sim E, Noble ME (2000). Structure of arylamine *N-*acetyltransferase reveals a catalytic triad. Nat Struct Biol.

[54] Fullam E, Westwood IM, Anderton MC, Lowe ED, Sim E, Noble ME (2008). Divergence of cofactor recognition across evolution: coenzyme A binding in a prokaryotic arylamine *N-*acetyltransferase. J Mol Biol.

[55] Abuhammad A, Lowe ED, McDonough MA, Shaw Stewart PD, Kolek SA, Sim E, Garman EF (2013). Structure of arylamine *N-*acetyltransferase from *Mycobacterium tuberculosis* determined by cross-seeding with the homologous protein from *M. marinum*: triumph over adversity. Acta Crystallogr D Biol Crystallogr.

[56] Szakacs Z, Noszal B (2006). Determination of dissociation constants of folic acid, methotrexate, and other photolabile pteridines by pressure-assisted capillary electrophoresis. Electrophoresis.

[57] Brown HC, McDaniel DH, Haflinger P (1955). Determination of Organic Structures by Physical Methods.

[58] Nobilis M, Vybiralova Z, Sladkova K, Lisa M, Holcapek M, Kvetina J (2006). High-performance liquid-chromatographic determination of 5-aminosalicylic acid and its metabolites in blood plasma. J Chromatogr A.

[59] Lukács M, Barcsa G, Kovács-Hadady K (1998). The effects of pH, ionic strength and buffer concentration of mobile phase on R F of acidic compounds in ion-pair TLC. Chromatographia.

[60] Machado JD, Gomez JF, Betancor G, Camacho M, Brioso MA, Borges R (2002). Hydralazine reduces the quantal size of secretory events by displacement of catecholamines from adrenomedullary chromaffin secretory vesicles. Circ Res.

[61] Becker C, Dressman JB, Amidon GL, Junginger HE, Kopp S, Midha KK, Shah VP, Stavchansky S, Barends DM (2007). Biowaiver monographs for immediate release solid oral dosage forms: isoniazid. J Pharm Sci.

[CR62] The pre-publication history for this paper can be accessed here: http://www.biomedcentral.com/2050-6511/15/68/prepub

