# Supplementary figures and images for: Differences between murine arylamine N-acetyltransferase type 1 and human arylamine N-acetyltransferase type 2 defined by substrate specificity and inhibitor binding
Source: BMC Pharmacol Toxicol. 2014 Nov 29;15:68. doi: 10.1186/2050-6511-15-68 (PMC4258814; doi:10.1186/2050-6511-15-68)

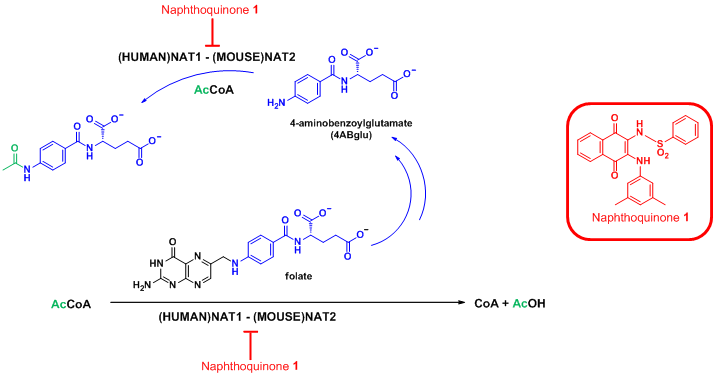

Supplement: Supplementary file 1 — Additional file 1: Figure S1: Selective functionality of (HUMAN)NAT1*4 and (MOUSE)NAT2*1. Two catalytic reactions are selective to (HUMAN)NAT1*4 and (MOUSE)NAT2*1 amongst mammalian NATs: the N-acetylation of the folate catabolite 4ABglu and the folate-dependent hydrolysis of AcCoA. Both reactions are selectively inhibited by naphthoquinone 1 (shown in red). (TIFF 55 KB) [file 40360_2014_351_MOESM1_ESM.tiff]

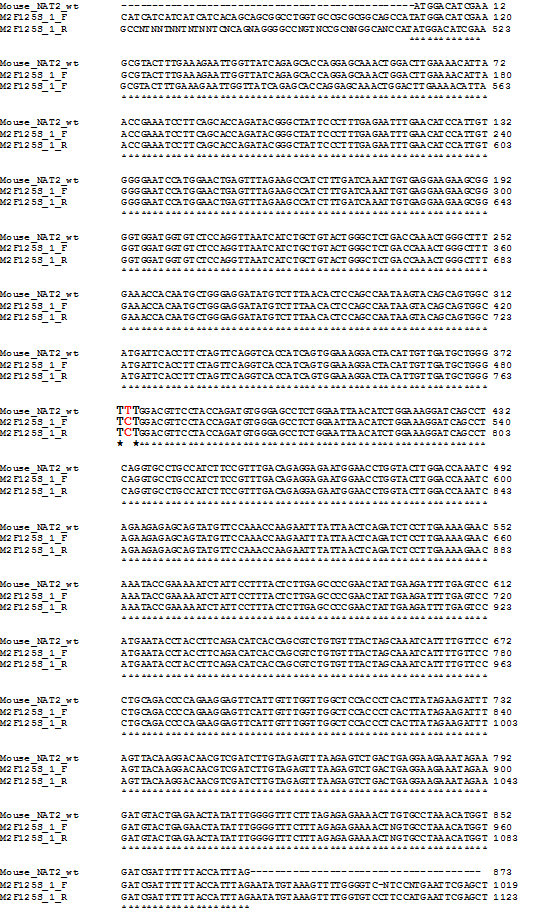

Supplement: Supplementary file 2 — Additional file 2: Figure S2: Multiple sequence alignment of (MOUSE)Nat2*1 gene and (MOUSE)Nat2_F125S gene. Alignment of (MOUSE)Nat2*1 sequence and the forward and reverse sequences obtained after mutagenesis was conducted by ClustalW [42]. The single mutated nucleotide is highlighted in red. *indicates nucleotide identity among all three genetic sequences. No additional mutations were generated during the process of site directed mutagenesis. (TIFF 130 KB) [file 40360_2014_351_MOESM2_ESM.tiff]

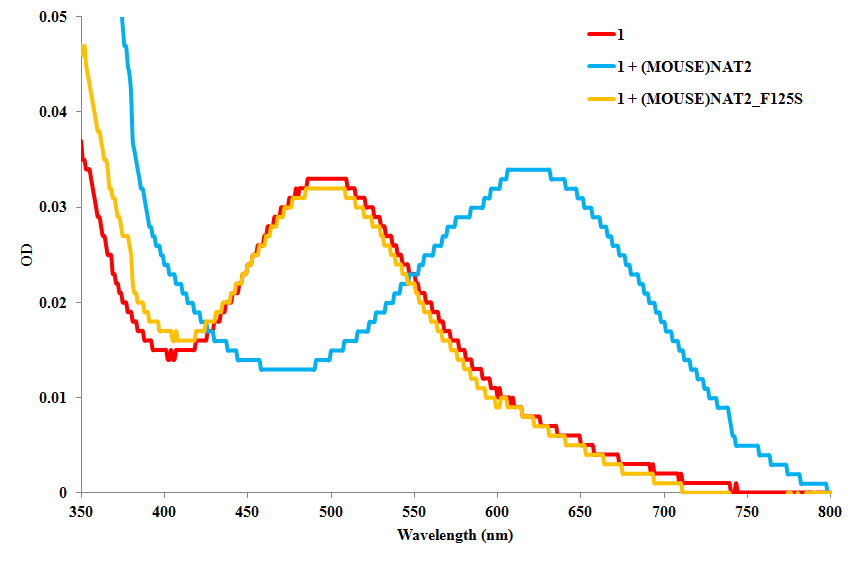

Supplement: Supplementary file 4 — Additional file 4: Figure S3: Visible spectra of naphthoquinone 1 in the presence of different mammalian NAT variants. Naphthoquinone 1 (15 μM) was incubated with 20 mM Tris–HCl, pH 8.0, 5% DMSO (v/v) (red line) or NAT variants (30 μM): (MOUSE)NAT2*1 (blue line); (MOUSE)NAT2_F125S (yellow line)). Wavelength scans from 800 to 350 nm were recorded against the appropriate blank (20 mM Tris–HCl, pH 8.0, 5% DMSO (v/v)). (TIFF 71 KB) [file 40360_2014_351_MOESM4_ESM.tiff]

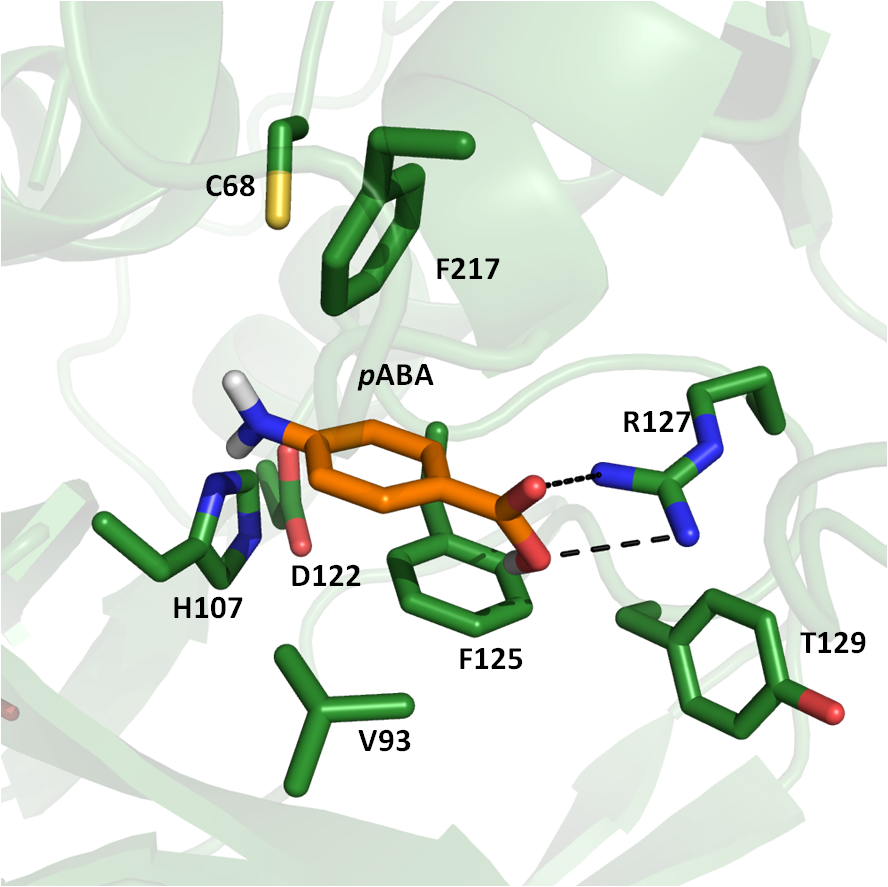

Supplement: Supplementary file 6 — Additional file 6: Figure S4: Substrate binding pockets of (HUMAN)NAT1*4 with 4ABA docked. Maximised view of the active sites of (HUMAN)NAT1*4 with the arylamine substrate 4ABA docked. The overall structure of (HUMAN)NAT1*4 is drawn in ribbon diagram (green) [PDB:2PQT]. The side chain of the key residues involved in substrate binding within the active site are drawn in stick representation and labelled with carbon atoms in the colour of the enzyme, nitrogen in blue, oxygen in red and sulphur in yellow. The arylamine substrate 4ABA is labelled with carbon atoms in orange, nitrogen in blue, oxygen in red and polar hydrogen in gray. The figures were generated using PyMOL [41]. (TIFF 421 KB) [file 40360_2014_351_MOESM6_ESM.tiff]
