# Supplementary material for: Differences between murine arylamine N-acetyltransferase type 1 and human arylamine N-acetyltransferase type 2 defined by substrate specificity and inhibitor binding
Source: BMC Pharmacol Toxicol. 2014 Nov 29;15:68. doi: 10.1186/2050-6511-15-68 (PMC4258814; doi:10.1186/2050-6511-15-68)
Supplement: Supplementary file 3 — Additional file 3: Table S1: Chemical NAT substrates used in this study. Ionic charges of substrates at assay pH 8.0 are shown according to their pKa(H) values [56–61]. (DOCX 63 KB) [file 40360_2014_351_MOESM3_ESM.docx]

**Supplementary Table 1**

**Chemical NAT substrates used in this study**

Ionic charges of substrates at assay pH 8.0 are shown according to their pK_a(H)_ values [[60-65](#_ENREF_60)].

| **Physico-chemical group** | **Subgroup** | **NAT substrate** | **Chemical structure**  **at pH 8.0** | **pK_a(H)_ values and References** |
| --- | --- | --- | --- | --- |
| ***arylamines with negatively charged substituents at pH 8.0*** | folate catabolites | **4ABglu** |  | 2.7 (N*H*_3_^+^)  3.6 (α-COO*H*)  4.7 (γ-COO*H*) |
|  |  | **4ABA** |  | 2.4 (N*H*_3_^+^)  4.9 (COO*H*) |
|  | salicylic acids | **4AS** |  | 2.3 (N*H*_3_^+^)  6.0 (COO*H*)  13.2 (O*H*) |
|  |  | **5AS** |  | 3.0 (N*H*_3_^+^)  6.0 (COO*H*)  13.9 (O*H*) |
| ***arylamines with neutral electron-rich functional substituents*** | halogenated arylamines | **4CA** |  | 3.8 (N*H*_3_^+^) |
|  |  | **4BA** |  | 3.4 (N*H*_3_^+^) |
|  |  | **4IA** |  | 3.8 (N*H*_3_^+^) |
|  | alkyloxy- and aryloxy-substituted arylamines | **ANS** |  | 5.2 (N*H*_3_^+^) |
|  |  | **4AV** |  | 4.7 (N*H*_3_^+^) |
|  |  | **HOA** |  | 5.1 (N*H*_3_^+^) |
|  |  | **POA** |  | 4.36 (N*H*_3_^+^) |
|  | other arylamine | **SMZ** |  | 2.4 (N*H*_3_^+^) 7.4 (sulfonamide) |
| ***Hydrazines*** | | **INH** |  | 2.0 (N*H*_2_^+^-NH_3_^+^)  3.9 (NH-N*H*_3_^+^)  10.8 (pyridine) |
|  |  | **HDZ** |  | 7.3 (NH-N*H*_3_^+^ |
