# Supplementary material for: Differences between murine arylamine N-acetyltransferase type 1 and human arylamine N-acetyltransferase type 2 defined by substrate specificity and inhibitor binding
Source: BMC Pharmacol Toxicol. 2014 Nov 29;15:68. doi: 10.1186/2050-6511-15-68 (PMC4258814; doi:10.1186/2050-6511-15-68)
Supplement: Supplementary file 5 — Additional file 5: Table S2: Comparison of eukaryotic NAT sequence identity and similarity. Percentage identity (no shade) and similarity (grey shade) values were calculated amongst five mammalian NATs using BLAST2 sequences. (DOCX 12 KB) [file 40360_2014_351_MOESM5_ESM.docx]

**Supplementary Table 2.**

**Comparison of eukaryotic NAT sequence identity and similarity**

Percentage identity (no shade) and similarity (grey shade) values were calculated amongst five mammalian NATs using BLAST2 sequences.

|  | **(HUMAN)**  **NAT1*4** | **(MOUSE)**  **NAT2*1** | **(MESAU)**  **NAT2*1** | **(MOUSE)**  **NAT1*1** | **(HUMAN)**  **NAT2*4** |
| --- | --- | --- | --- | --- | --- |
| **(HUMAN)NAT1*4** |  | 82 | 81 | 74 | 71 |
| **(MOUSE)NAT2*1** | 94 |  | 93 | 81 | 67 |
| **(MESAU)NAT2*1** | 91 | 96 |  | 82 | 61 |
| **(MOUSE)NAT1*1** | 85 | 89 | 88 |  | 64 |
| **(HUMAN)NAT2*2** | 88 | 77 | 75 | 72 |  |
